# Marjolin’s Ulcer in Laron Syndrome - an Unexpected Combination: A Case Report

**DOI:** 10.5704/MOJ.2003.012

**Published:** 2020-03

**Authors:** EM de la Paz

**Affiliations:** Department of Orthopaedics, The Medical City Clark, Angeles City, Philippines

**Keywords:** Marjolin’s ulcer, Laron syndrome, IGF-1 deficiency, cancer protection, below knee amputation

## Abstract

Marjolin’s ulcer is an atypical malignancy that develops from deep scars of chronically traumatised skin. Laron syndrome (LS) is a rare autosomal recessive growth retardation from a mutation in the growth hormone receptor (GHR) gene leading to defective GHR, growth hormone insensitivity and eventual low levels of insulin-like growth factor type 1 (IGF-1). Affected individuals present with proportionate dwarfism and other characteristic physical defects, but at the same time are conferred protection against cancer due to low serum levels of IGF-1. We report an exceptional case of Marjolin’s ulcer in the foot of a female LS patient 30 years after she sustained flame burns as a 6-month-old baby. Three months before coming to us, she had a 2x3cm ulcer that turned into a rapidly enlarging fungating mass involving the leg, ankle, and foot. Histopathologic analysis of an incision biopsy showed well-differentiated squamous cell carcinoma. The extent of her lesion precluded wide excision. Below knee amputation was done. A second biopsy confirmed the histopathologic diagnosis. This is the first reported case in the literature of Marjolin’s ulcer in LS which raises the possibility that IGF-1 deficiency does not completely protect against squamous cell cancer.

## Introduction

Marjolin’s ulcer is an atypical malignant degeneration, usually to squamous cell carcinoma^1^, of chronic deep scar tissue from previously traumatised or burned skin. Laron syndrome (LS) is a rare autosomal recessive dwarfism caused by a mutation in the growth hormone receptor gene. First described in 1966 by Laron *et al*^2^, there are only over three hundred cases identified worldwide. The growth hormone (GH) insensitivity and IGF-1 deficiency that causes characteristic physical deformities also reduces susceptibility to cancer^3^.

We present a case of a female with LS that developed Marjolin’s ulcer from a burn wound on the foot three decades after injury. Previously treated by various health care practitioners, she was referred by a burn care nurse specialist. The diagnostic delay, the decision that led to and outcome of treatment of this very unexpected combination of two rare conditions are described.

## Case Report

A 30-year-old female was referred for a fungating mass on the left heel. At six months old, she sustained flame burns from a kerosene lamp on her left upper and lower extremities. The wounds were left to heal secondarily resulting in extensive scarring and contractures of the hand, foot, ankle and distal leg. Two years ago, a blister appeared on the heel enlarging to a 1cm diameter ulcer from incessant scratching. A burn care nurse practitioner helped care for the wound with medical-grade honey, resulting to complete healing at times. An ulcer about 2x3cms on the same site that developed just three months ago exacerbated despite frequent wound care. This rapidly expanded to a fungating mass that caused difficulty in walking and significant pain at rest. One month ago, an incision biopsy was done in another hospital. Histopathological analysis showed squamous cell carcinoma (well-differentiated, grade I).

She is obese with short, but proportionate, stature at only 119cm high (height standard deviation score: <-4.4). She has underdeveloped mandible, depressed nasal bridge, prominent forehead and bluish sclerae. Hairline showed frontotemporal recession. She speaks in a very high-pitched voice, has crowding of defective teeth and infantile genitalia. Random GH level was 15ng/mL (normal: 1-14 ng/ml), and IGF-1 was 25ng/mL (normal: >109-284ng/mL). Genetic analysis, GH stimulation test, and insulin-like growth factor binding protein-3 measurement could not be performed for this patient. Based on these clinical features, a diagnosis of LS was made.

**Fig. 1:**
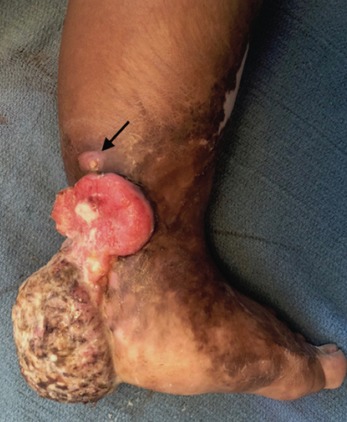
Medial view of the foot. Measuring 6x10x12cm at its widest dimensions, the fungating mass extends from 6cm cephalad to the malleoli to the plantar aspect of the heel. It started as a recurrent blister at the heel similar to the lesion indicated by the arrow. Burn scars involve the entire foot and distal leg.

**Fig. 2:**
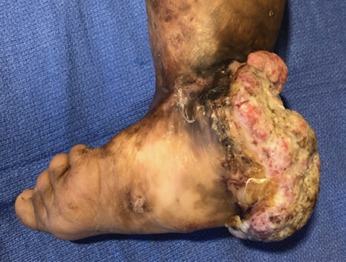
Lateral view of the foot. Involving the entire heel and the insertion of the tendon of Achilles, the ulcerating mass is foul-smelling with beginning necrosis in some areas. Rapid growth is noticeable at its most proximal and medial margin. Scarring affects the entire foot, ankle, and distal leg. The forefoot is abducted and everted due to the contractures. Post-burn bent contracture deformities are evident on the 2nd-5th toes.

The mass measured 6x10x12cm at its widest dimensions, predominantly posterior on the heel extending from 6cm cephalad to the malleoli to the plantar aspect of the heel (Fig. 1 and 2). It was foul-smelling with beginning areas of necrosis on the heel. The proximal border of the lesion, reddish and fleshy, displayed the fastest growth with some bleeding on the edge. The rest of the entire foot are scarred. She had no palpable lymph nodes.

The extent of the lesion precluded wide excision. She consented to a below knee amputation which was done. The distal end of the posterior skin flap by the Burgess technique was about 3cm from the most proximal edge of the lesion. Biopsy done on the amputated leg confirmed squamous cell carcinoma with margins negative for malignant cells. She was informed about the possible development of Marjolin’s ulcer on her left hand and advised regular inspection of the rest of her scars. The importance of periodic examination of her lymph nodes was also emphasised, thus the need for regular follow-up in her lifetime.

## Discussion

Because the pituitary gland is uninvolved, the GH level in LS is normal or sometimes elevated due to negative feedback to the gland^3^. IGF-1however is low as its production in the liver is dependent on GH stimulation. With the preponderance of clinical features, the normal GH and low IGF-1 levels, we surmised our patient has LS. The link of IGF-1 to cancer risk is well stablished, having an association with the oncogenesis of both solid and haematologic malignancies^4^. Due to IGF-1 deficiency, LS confers a level of protection against cancer^5^. Twice done, biopsy confirmed squamous cell carcinoma in our patient. Marjolin’s ulcer is known to be an aggressive skin cancer developing in deep burn wounds allowed to heal secondarily. After a latency of three decades, our patient developed a recurrent wound in the last two years followed by a rapidly growing mass just within three months from its frank ulcerative stage. It can be said that in this patient, the aggressiveness of the Marjolin’s ulcer subjugated the protective effect of LS against the development of squamous cell carcinoma. We have not found a similar report of cutaneous cancer developing in LS. Reports in the literature of any type of cancer occurring in LS are sparse. Indeed, in a cohort of 230 LS patients followed by the group of Dr. Laron up to the age of 85, none developed cancer^3^. In an Ecuadorian cohort of 99 LS patients, only one developed cancer, a papillary serous epithelial ovarian tumour^5^.

While wide excision is the most commonly applied treatment for Marjolin’s ulcer^1^, the extent of the lesion in our patient precluded this option. Performing a wide excision would have left a defect too difficult to resurface. It would have also entailed sacrificing the TA from its insertion and a portion of the gastrocsoleus together with neurovascular structures, rendering an already deformed foot more dysfunctional. Her small stature and some delay in the diagnosis may have contributed to limb sacrifice as the tumour covered more area relative to what it would have in an adult of normal or bigger stature in the same period.

Marjolin’s ulcer can occur in Laron syndrome. Delay in diagnosis of this aggressive skin cancer in an individual of small stature may render an otherwise resectable tumour amenable only to amputation. Carcinoma should be considered even in IGF-1 deficiency states, unless proven otherwise, in chronic deep burn wounds that suddenly develop a rapidly enlarging ulcer after a long period of latency.
